# Mapping Cancer for Community Engagement

**Published:** 2008-12-15

**Authors:** Kirsten M. M. Beyer, Gerard Rushton

**Affiliations:** Dept of Geography, University of Iowa; University of Iowa, Iowa City, Iowa

## Abstract

**Introduction:**

Two research strategies may reduce health disparities: community participation and the use of geographic information systems. When combined with community participation, geographic information systems approaches, such as the creation of disease maps that connect disease rates with community context, can catalyze action to reduce health disparities. However, current approaches to disease mapping often focus on the display of disease rates for political or administrative units. This type of map does not provide enough information on the local rates of cancer to engage community participation in addressing disparities.

**Methods:**

We collaborated with researchers and cancer prevention and control practitioners and used adaptive spatial filtering to create maps that show continuous surface representations of the proportion of all colorectal cancer cases diagnosed in the late stage. We also created maps that show the incidence of colorectal cancer.

**Results:**

Our maps show distinct patterns of cancer and its relationship to community context. The maps are available to the public on the Internet and through the activities of Iowa Consortium for Comprehensive Cancer Control partners.

**Conclusion:**

Community-participatory approaches to research are becoming more common, as are the availability of geocoded data and the use of geographic information systems to map disease. If researchers and practitioners are to engage communities in exploring cancer rates, maps should be made that accurately represent and contextualize cancer in such a way as to be useful to people familiar with the characteristics of their local areas.

## Introduction

Cancer health disparities, defined as "differences in cancer incidence (new cases), cancer prevalence (all existing cases), cancer death (mortality), cancer survivorship, and burden of cancer or related health conditions that exist among specific population groups" (1), are a priority for public health research. Cancer is the second leading cause of death in the United States (2), killing approximately 550,000 people per year (3). Rates of cancer incidence, mortality, and survival, as well as measures of access to resources for treatment and prevention, can in some cases be correlated with race, ethnicity, socioeconomic status (SES), and geographic location (4,5). Consequently, interest in cancer disparities is growing.

Materials that guide comprehensive cancer control activities do not address spatial variation in cancer prevalence (6). This absence of a focus on the geographic dimension of cancer prevention and control is evident elsewhere. For example, a detailed framework for comprehensive cancer control and prevention (7) makes no mention of geography. To develop a deeper understanding and more effectively target prevention and control activities, each of the 4 phases of that framework could include a geographic component: geographic gap analysis, geographic basis of possible and feasible strategies, geographic interventions, and geographic outcomes.

Two research strategies have been employed to understand and address disparities. First, as evidence of disparities has grown, some have called for a change in focus from uncovering variation to reducing disparities (8). There is a growing focus on translational research, which according to the National Cancer Institute, "transforms scientific discoveries arising from laboratory, clinical, or population studies into clinical applications to reduce cancer incidence, morbidity, and mortality" (9). Such research often incorporates community members, policy makers, practitioners, or other stakeholders in developing and investigating research questions. Participatory strategies, by empowering communities, can increase both the quality of research and the potential for research results to be translated into interventions to alleviate the problems identified (10-12).

Second, researchers are making more use of geographic information systems (GIS). GIS can uncover spatial variation in environmental exposures, social phenomena, and health outcomes, which will enable interventions to be targeted spatially. In addition, geocoded data are increasingly available, which makes GIS investigations increasingly possible. Applications of GIS in health include disease mapping, spatial epidemiology, and support for spatial decisions regarding provision of health care (13-15). GIS has value in the study of health disparities, particularly in its ability to connect data sets collected by different entities and organized at different levels of geography to illuminate difference on a fine scale, which is particularly of use in distinguishing communities' experiences. A report on approaches to health care disparities research listed such available data sets (16) but did not identify state cancer registries as a possible contributor to this task. Our work contributes to the development of methods for health disparities research by showing that a state cancer registry can be used to provide detailed geographic surveillance of cancer disparities.

Current approaches to disease mapping often make use of political or administrative borders, such as counties, to display disease rates. This type of mapping is problematic. It masks variation within units of area, gives the impression that a rate is static throughout the unit chosen, and often displays unstable disease rates in sparsely populated areas. A reliable spatial basis of support is needed to calculate and spatially represent disease rates (17,18).

We initiated a pilot mapping project to 1) build a collaboration between university researchers and cancer prevention and control practitioners and 2) open a dialogue about spatial patterns of cancer with the public by creating and distributing maps that accurately represent and contextualize the cancer burden.

## Methods

This project began with a collaboration between University of Iowa researchers and the Iowa Consortium for Comprehensive Cancer Control (ICCCC), which comprises stakeholders from various backgrounds, including researchers, legislators, and cancer survivors. The ICCCC works to develop and implement a comprehensive cancer control plan for Iowa (19). One of us (G.R.) joined the ICCCC Barriers to Screening Implementation Group, and at a meeting the group decided that mapping spatial variation in cancer incidence, late-stage cancer diagnosis, and cancer mortality would be especially valuable in cancer prevention and control efforts.

We proposed to create maps that represent rates of cancer as a continuously varying surface, as opposed to one divided by political or administrative boundaries. We would incorporate into those maps cues to the local context, enhancing the relevance to local communities. The maps would be distributed to the public via the World Wide Web and the activities of ICCCC. On the basis of the University of Iowa Institutional Review Board's recommendation, we withdrew our application for review because no human subjects were at risk.

### Data collection and preparation

We obtained cancer incidence data from the Iowa Cancer Registry through a data-sharing agreement. We received no individual identifiers with the data.

We obtained population data for Iowa zip code tabulation areas (ZCTAs) from the State Data Center of Iowa. The US Census Bureau discontinued tabulation of population information by zip code and began tabulating by ZCTA with the 2000 census. ZCTAs are generalized areal representations of zip codes and are built from census blocks. In calculating cancer incidence, population data for the ZCTA was used with cancer data for the zip code; thus, our incidence measurement is subject to spatial misalignment between zip code and ZCTA. This problem is partially controlled by aggregating the zip codes within the spatial filter areas (20). To simplify our discussion, we use "zip code" to describe analyses that make use of ZCTA data.

With these data, we created maps by using SAS software (SAS Institute Inc, Cary, North Carolina), Microsoft Excel spreadsheet software (Microsoft Corporation, Redmond, Washington), ESRI ArcMap software (Environmental Systems Research Institute, Inc, Redlands, California), and DMAP IV software (available for download at www.uiowa.edu/~gishlth/DMAP4/). To illustrate the steps taken and the results of our work, we focus on maps of colorectal cancer incidence and late-stage colorectal cancer diagnosis. It is widely argued that "tracking the rates of distant, or late, cancers is a good way to monitor the impact of cancer screening. When more cancers are detected in the early stages, fewer should be detected in the late stages" (21).

The cancer registry data set contained 12,615 cases of colorectal cancers newly diagnosed among Iowans from 1998 through 2003. In cases where 1 person was included more than once in the data set, we kept the record associated with the first tumor diagnosed. For the late-stage diagnosis maps, we excluded 1,314 records for which no stage information had been recorded.

We used the zip code as our level of geographic aggregation, as we believed it to be a complete and accurate geocode for cases in the Iowa Cancer Registry, and it provides sufficient local detail to be relevant to communities. Our final data set contained 5,576 colorectal cancer cases in men, representing 781 Iowa zip codes, and 5,725 cases in women, representing 728 Iowa zip codes. A total of 863 unique zip codes were represented in the final data set.

We then determined the number of expected cases for each zip code by using indirect age-sex standardization and the 2000 Iowa ZCTA population data with the following age groups, by years: 0-39, 40-44, 45-49, 50-54, 55-59, 60-64, 65-69, 70-74, 75-79, 80-84, and 85 or older. We used indirect rather than direct age adjustment because it applies the stable statewide rate to local populations, instead of applying local disease rates, which for small areas are unstable, to standard population weights. In addition, in communicating to map readers what they see, it is more effective to say that the map shows a comparison of the cases observed in the community with the number that would be expected if the community's rate were typical of the state, instead of saying that the map shows what the disease rate would be if the population characteristics of the community were typical of the state (which would be the explanation had direct adjustment been used). For each zip code, the expected number of colorectal cancer cases (or the number of late-stage diagnosis cases) of each cancer was computed by applying the statewide rates to the numbers of people (or cases) in each age-sex group in the zip code, for incidence (or proportion of late-stage diagnosis cases).

### Mapping

We mapped the ratio of observed cases to expected cases, showing how the rate of cancer incidence and late-stage detection varies across the state. Unfortunately, because of small numbers, we could not calculate rates adjusted by race or ethnicity, although disparities in cancer rates may exist for these groups and may warrant further investigation.

We created the maps by using adaptive spatial filtering (22,23) that was adapted from a fixed filter method (24). Adaptive spatial filtering is achieved by placing a grid of points over the study area and calculating the rate for each grid point by pulling in data from the surrounding area by using a circular filter until a threshold is met for the denominator. This method eliminates much of the instability in cancer rates that occurs because of differences in population sizes of areal units. A fixed filter would have resulted in less detail in urban areas or less stable rates in rural areas. Adaptive spatial filtering allowed us to achieve high geographic detail in urban areas as well as stable rates in rural areas.

To implement spatial filtering using aggregated data, we had to place our cancer data for each zip code at a single point within the zip code area. A common choice made by researchers is to use the centroid of an area, which is problematic for 2 reasons. First, a true centroid of a zip code cannot be determined because boundaries for zip code areas are not known. Second, using the centroid is not ideal because it is a representation of the center of land area in a zip code — not the center of population — and, thus, sometimes falls in an unpopulated location, influencing the spatial patterning of estimated rates. For each zip code, we selected the point location of the populated place that falls within the largest incorporated area in the corresponding zip code to represent the population. We obtained shapefiles of populated places and incorporated areas from a state geospatial data library (25). Where no incorporated area existed, we used the ZCTA centroid (20).

We joined the observed and expected numbers for each zip code to this set of point locations and calculated indirectly standardized rates for each grid point on a 3-mile grid. The size of the grid is relevant only to the degree that it is much finer than the geocode used for the data of interest — in this case, the zip code center. Our adaptive filter, centered on each grid point, expanded until it included at least 50 expected cancer cases as aggregated to the zip code level. We then interpolated the area in between grid points by using inverse distance weighting and the 8 nearest neighbors to create a continuous surface.

To make our maps meaningful in terms of local cancer rates, we made the following choices in producing our final maps. We overlaid our cancer burden layer at 50% transparency with a 1:100,000-scale US Geological Survey topographic map of Iowa, to allow topographic and other settlement information to be seen through the cancer layer. We also included geographically positioned names of major cities and highways to enhance the sense of place. For each map, we used a color scheme that transitioned from red to blue to indicate "hot" and "cold" spots — those that experienced rates higher and lower than the statewide rate, respectively. The 15 classes were determined by using quantile breaks; we used a large number of classes to present a smooth transition between categories and not give the impression of sharp changes in rates. With each map, we included a short description of the method used in its creation.

## Results

**Figure 1:**
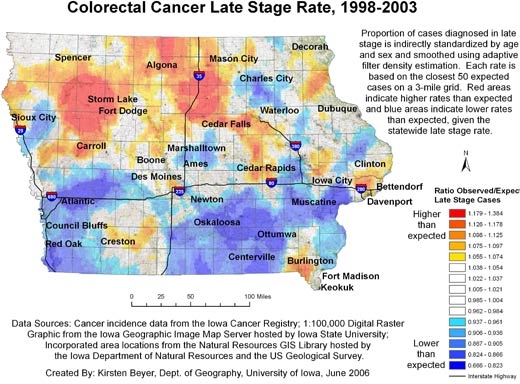
Example of map and explanatory information to illustrate the spatial pattern of the proportion of all colorectal cancer cases diagnosed in the late stage, mapped using adaptive spatial filtering, Iowa, 1998-2003. Data sources: Cancer incidence data from the Iowa Cancer Registry; 1:100,000 digital raster graphic from the Iowa Geographic Image Map Server hosted by Iowa State University; incorporated area locations from the Natural Resources GIS Library hosted by the Iowa Department of Natural Resources and the US Geological Survey.

**Figure 2:**
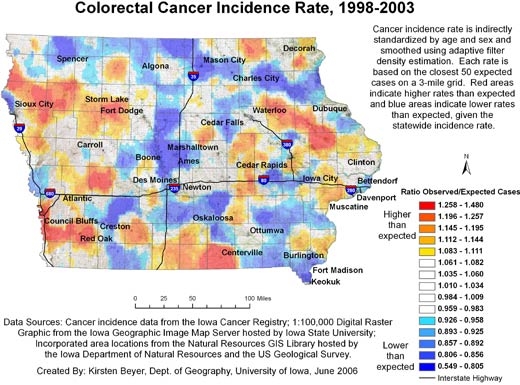
Example of map and explanatory information to illustrate the spatial pattern of colorectal cancer incidence, mapped using adaptive spatial filtering, Iowa, 1998-2003. Data sources: Cancer incidence data from the Iowa Cancer Registry; 1:100,000 digital raster graphic from the Iowa Geographic Image Map Server hosted by Iowa State University; incorporated area locations from the Natural Resources GIS Library hosted by the Iowa Department of Natural Resources and the US Geological Survey.

Figures 1 and 2 show our final maps of late-stage diagnosis of colorectal cancer and incidence of colorectal cancer, respectively. The figures also include brief explanatory information about the data. There are observable spatial patterns in both colorectal cancer incidence and late-stage diagnosis rates. Late-stage colorectal cancer diagnosis exhibits a north-south gradient, with higher rates of cancers diagnosed in the late stage in the north. Colorectal cancer incidence and the proportion of cancers diagnosed in the late stage as calculated for 6,300 grid points are not strongly correlated (*r* = −0.0688). Some places in the state experience high or low rates of both incidence and late-stage diagnosis. The area around Storm Lake in northwest Iowa, for instance, experiences elevated rates of both incidence and late-stage colorectal cancer. These elevated rates could be related to the fact that this community has a high percentage of minority populations, in particular the Tai Dam/Lao and Hispanic communities; 21.1% of Storm Lake residents identify themselves as Hispanic or Latino, and 7.8% identify as Asian (26).

**Table:** 

**Table:** 

**Figure 3:**
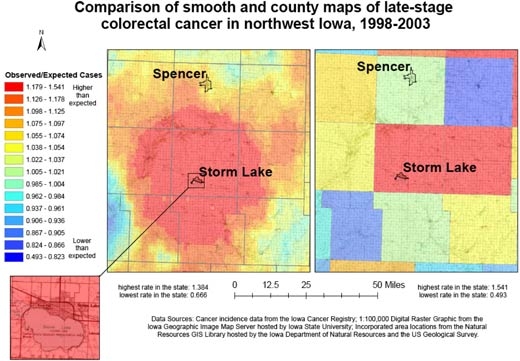
Example of map and explanatory information to illustrate the comparison of adaptive spatial filtering and traditional mapping using county boundaries for 9 northwest Iowa counties to show all colorectal cancer cases diagnosed in the late stage, 1998-2003. Data sources: Cancer incidence data from the Iowa Cancer Registry; 1:100,000 digital raster graphic from the Iowa Geographic Image Map Server hosted by Iowa State University; incorporated area locations from the Natural Resources GIS Library hosted by the Iowa Department of Natural Resources and the US Geological Survey.

Figure 3 compares the continuously varying map with a county map for the prevalence of late-stage colorectal cancer for 9 northwest Iowa counties. Both maps use the same legend, based on the 15 quantile breaks in the spatially filtered map, and include the high and low values of the county rate map because it had a wider range. In the continuously varying map, topographic detail is observable beneath the layer displaying cancer data, orienting the map reader to community context, and the cancer rates can be observed at the sub-county level. In addition, a distinct spatial pattern of high late-stage rates centered on Storm Lake is observable. This representation of cancer rates can be contrasted with the county map, which was created with data aggregated to the county level and masks variation within counties as well as the larger spatial pattern.

**Table:** 

**Figure 4:**
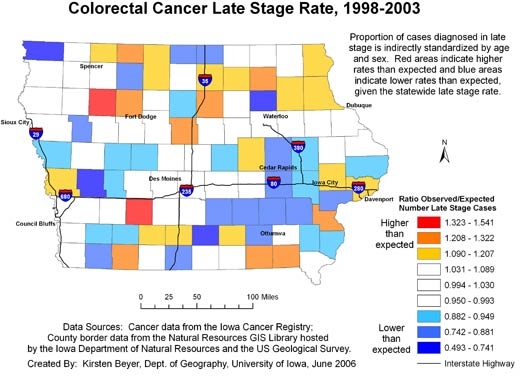
Example of map and explanatory information to illustrate the spatial pattern of the proportion of all colorectal cancer cases diagnosed in the late stage, mapped using county boundaries, Iowa, 1998-2003. Data sources: Cancer incidence data from the Iowa Cancer Registry; county border data from the Natural Resources GIS Library hosted by the Iowa Department of Natural Resources and the US Geological Survey.

In addition, the continuously varying map provides a more reliable picture of actual rates of cancer at the local level. The area surrounding Storm Lake in northwest Iowa has a high rate of late-stage colorectal cancer diagnosis, but when we examine the state by county (Figure 4), we see that the Storm Lake area is still identified as having a high rate (Buena Vista County, containing Storm Lake, has the second highest county rate in Iowa), but Adair County southeast of Des Moines is identified as having the highest rate. While the late-stage rate for Buena Vista County was calculated based on 38.63 expected and 58 observed cases, the rate for Adair County was based on only 11.68 expected cases and 18 observed cases. In Figure 2, we see that the elevated rate in Adair County has disappeared. If the county map were used to determine which counties to target for intervention programs to promote colorectal cancer screening efforts, without a consideration of rate instability, efforts would be misguided.

**Table:** 

a Fewer than 10 expected late-stage cases.

The maps are distributed to the public through both the Internet and the ICCCC partners. The maps are posted on a Web site hosted by the University of Iowa (www.uiowa.edu/~gishlth/ICCCCMaps/) with a narrative report and a guide to interpretation. Several maps were used in a direct community interaction organized by the Iowa Cancer Registry. This event, "Cancer in Your Community," took place in Sioux City, Iowa, on September 21, 2006, drawing approximately 130 people. Dr Charles Lynch, director of the Iowa Cancer Registry and presenter at the event, noted that the spatially filtered maps were especially useful in his interaction with the public, as people come to an event such as his with an understanding of their geographic area, and seeing the local detail presented in the map helps the audience connect cancer rates with knowledge of their local areas, "pointing them in directions that they can take to alleviate problems they see" (C. Lynch, MD, MS, PhD, oral communication, November 2006).

ICCCC partners gave the maps a positive reception at the ICCCC meeting in Des Moines, Iowa, on October 12, 2006, and the maps were featured in the July/August 2006 ICCCC newsletter (27). Jolene Carver, Iowa Case Management Coordinator at the Iowa Department of Public Health for the Breast and Cervical Cancer Early Detection Program and ICCCC member, said that her program had developed an outreach program to target 2 counties in Iowa using our maps of breast cancer. She said, "An outreach worker was contracted to promote education to women who live in these 2 counties. This education will encourage regular screening for breast cancer using mammograms and clinical breast exams" (J. Carver, Iowa Department of Public Health, e-mail communication, April 2008). The Barriers to Screening Implementation Group of ICCCC has also used the maps to determine which areas in the state to target for a colorectal cancer screening education campaign. Other ICCCC partners have expressed interest in using the maps for prevention and control work and in continuing the collaboration and mapping efforts.

## Discussion

The difference between the maps created in this research and those traditionally produced to represent cancer rates by county are relevant to community participation. The continuously varying maps, in combination with the visualization of local context, provide local detail that cannot be achieved by using maps with large geographic units for displaying rates. These maps based on local data make the information about cancer rates more meaningful to people who are familiar with the local area. ICCCC partners have begun to use these maps in their work, and the collaboration continues.

This collaborative project is a start for research that integrates community participation and GIS for cancer prevention and control. Many issues must be considered in future work, including the tension between community involvement and health data confidentiality constraints, the structure of partnerships, the role of GIS, the potential for community-generated data and incorporation of local knowledge of "place," the potential for use of interactive computer- or Web-based mapping applications, the definition of terms such as *community* and *neighborhood* for purposes of participation as well as data analysis, pitfalls of map interpretation, and the many cartographic choices that must be made. In addition, because we did not conduct a systematic assessment of our maps, future work could more systematically evaluate the degree to which these maps improve on traditional mapping by areal units from the perspective of cancer prevention and control practitioners and the public.

As noted in the ICCCC newsletter, "Interpretation of this data will be the next step. For example, it will be important to determine if incidence rates are high in some areas due to increased screening or if rates are higher as a result of other factors" (27). Direct involvement of affected communities in generating and interpreting geographic information will improve our ability to address these questions.

A limitation identified in the literature that should be noted in working with spatially adaptive filter maps and the public is the notion that rates are calculated for varying sizes of areas, and thus the spatial resolution is not consistent across the map (13,22). Thus, if rates are high in a low-population area, that rate may have been calculated with data from a larger expanse of land. This is a central message that must be communicated. Maps of this nature may also benefit from being presented in concert with information about statistical significance (22). For the purposes of this project, we did not want to overburden the map reader with information about statistical significance.

Indirectly adjusted rates have the limitation that comparisons across areas apply a different set of weights to each area, which reflects the age distribution of the area in question. However, for resource allocation purposes, the difference between actual and expected numbers of late-stage cancer cases is a measure of the need for additional resources such as screening services.

By creating this initial set of maps and obtaining feedback from ICCCC partners and the public, we begin a dialogue about the types of questions communities are interested in and potential problems of interpreting the maps, and we can direct our attention to the development of tools and strategies that will allow us to address questions of interest and overcome problems. This mapping project is a beginning for further efforts to communicate and work with the public to alleviate cancer through efforts to change behaviors, investigate environmental exposures, and address disparities. The central goals of future work must be to improve scientific understanding of cancer rates and to promote effective, informed, and targeted intervention, involving the public in investigating community burdens of cancer at an appropriate scale and bringing the knowledge gained from this investigation into prevention and control activities.
